# Impact of Partial Oil Removal on Energy Content of Distillers Grains Plus Solubles for Finishing Cattle

**DOI:** 10.3390/ani14162329

**Published:** 2024-08-12

**Authors:** Jordan E. Burhoop, Jessica L. Sperber, Curt J. Bittner, F. Henry Hilscher, Jim C. MacDonald, Galen E. Erickson

**Affiliations:** Department of Animal Science, University of Nebraska-Lincoln, Lincoln, NE 68583-0908, USAjsperber2@unl.edu (J.L.S.); jmacdonald2@unl.edu (J.C.M.)

**Keywords:** corn oil, de-oiling, digestibility, finishing cattle

## Abstract

**Simple Summary:**

Partial removal of corn oil from corn-based distillers grains plus solubles is becoming commonplace since introduced in 2012. As a result of partial oil removal, distillers grains’ fat content has decreased from an average of 12 to 13% down to an average of 6 to 9% on a DM basis, with most ethanol plants producing corn-based distillers grains with an average of 8 to 9% corn oil today. There remains the question of the impact of partial oil removal from distillers grains plus solubles on energy value as a feedstuff for finishing beef cattle. Performance and digestion experiments were performed comparing four treatments, including a full-fat distillers grains treatment, a partial oil removal distillers grains treatment referred to as “de-oiled”, a treatment with corn oil added to de-oiled distillers grains, and a dry-rolled corn control. Including modified distillers grains improved feed efficiency by 6 to 11% across treatments when compared to feeding dry-rolled corn. Adding 2% corn oil to de-oiled distillers grains improved efficiency by 4.9% compared to feeding de-oiled distillers grains. Adding distillers grains regardless of treatment type improved the digestible energy consumed by the animal despite decreasing organic matter digestibility. Fiber digestibility improved with diets containing distillers grains, but was slightly hindered when oil was added as an ingredient. The impact of feeding de-oiled distillers grains with 8.9% fat was negligible in terms of cattle performance compared to full-fat distillers grains with 11.6% fat in diets containing 40% distillers grains on a DM basis.

**Abstract:**

Two experiments evaluated the impact of the reduction in the percentage of corn oil remaining in distillers grains plus solubles (DGS) after the ethanol plant de-oiling process or by adding corn oil back to DGS following de-oiling on finishing cattle performance and nutrient digestion. Experiment 1 utilized 320 yearling steers (initial BW = 413 kg; SD = 25 kg) fed in 32 pens (10 steers/pen) and assigned to one of four treatments (n = 8 pens/treatment). The four treatments consisted of a blended DRC:HMC corn control diet (CON), de-oiled modified distillers grains plus solubles included at 40% of diet DM (DODGS), de-oiled modified distillers grains plus solubles included at 38% of diet DM plus 2% added corn oil (DODGS + Oil), and full-fat modified distillers grains plus solubles included at 40% of diet DM (FFDGS). The DODGS product contained 8.9% fat while the FFDGS product contained 11.6% fat. Dry matter intake (DMI) was impacted by treatment (*p* = 0.01) with steers fed DODGS having the greatest DMI and steers fed CON, DODGS + Oil, and FFDGS having lower DMI. Dietary treatment tended to impact ADG (*p* = 0.06) with steers fed DODGS and DODGS + Oil having greater gains than CON, with FFDGS being an intermediate. As a result of increased ADG, G:F differed between treatments (*p* < 0.01) with the greatest feed efficiency observed for steers fed DODGS + Oil and FFDGS. Including MDGS in the diet improved G:F by 6 to 11% compared to feeding DRC:HMC corn blend, with an improvement in G:F of 4.9 and 1.2% for DODGS + Oil and FFDGS, respectively, compared to DODGS. Hot carcass weight was impacted by dietary treatment (HCW; *p* = 0.05), with DODGS- and DODGS + Oil-fed steers having the heaviest HCW, CON steers having the lightest HCW, and FFDGS being an intermediate. Experiment 2 was a 5 × 4 unbalanced Latin rectangle digestion experiment with four diets, five ruminally cannulated steers, and five periods that utilized the same treatments as Exp. 1. Dietary fat measured 4.2, 6.0, 7.9, and 7.1% for CON, DODGS, DODGS + Oil, and FFDGS, respectively. Intakes of DM, OM, and energy as well as total tract fat digestibility and DE (Mcal/d) were not impacted by dietary treatment (*p* ≥ 0.46). When corn oil was added back to de-oiled MDGS, there was a negative impact on digestibility of OM (*p* < 0.01) and NDF (*p* = 0.07) compared with DODGS, FFDGS, and CON. Partially removing oil from modified distillers grains plus solubles did not significantly impact cattle performance, carcass traits, energy content, or digestibility when MDGS was included at approximately 40% of diet DM.

## 1. Introduction

Distillers grains plus solubles (DGS) are a corn-based feedstuff created as a byproduct of ethanol production. Distillers grains are commonly fed in US finishing diets as either a protein source, when included in the diet up to 15% of dietary DM, or as an energy source, when included in the diet greater than 15% of dietary DM. The ethanol industry has recently started removing components of distillers grains, such as corn oil, which changes the nutrient composition of DGS. Changing the nutrient composition by de-oiling DGS, specifically for energy content which is provided by oil in the germ of the grain, may have a negative impact on finishing cattle performance. One proposed method to overcome the loss in energy from de-oiling DGS is to add corn oil (or other fat products) back to the diet to ensure that cattle performance is not hindered, in terms of gain and feed efficiency [1].

Previous literature has compared de-oiled DGS to normal-fat DGS and their impact on cattle performance. Jolly-Breithaupt et al. [2] compared de-oiled versus normal-fat modified distillers grains plus solubles (MDGS) at 40% diet DM inclusion, and reported no differences in cattle performance traits due to the fat content of MDGS. Another study compared de-oiled versus normal-fat wet distillers grains plus solubles (WDGS) at increasing concentrations, and observed an increase in DMI for de-oiled WDGS; however, oil content did not impact final BW, ADG, or G:F [2]. Vander Pol et al. [3] fed WDGS (0, 20, or 40% DM inclusion) or corn oil at three different inclusions (0, 2.5, or 5.0%). Cattle fed 5.0% corn oil had worsened overall performance than cattle fed all other diets. The results of Vander Pol et al. [3] demonstrated an improvement in cattle performance with normal-fat WDGS, with a reduction in G:F for cattle fed corn oil treatments, suggesting that the fat in the two products is different. A metabolism experiment was conducted to evaluate the digestibility of WDGS compared with corn fiber and corn oil in finishing diets [3]. Cattle fed WDGS had a lower rumen pH, greater propionate, lower acetate/propionate ratio, and greater total tract fat digestion compared to cattle fed corn fiber and corn oil. Bremer [1] concluded that cattle fed WDGS had the lowest total tract DM and fat digestibility, while cattle fed corn oil had the lowest total tract NDF digestibility. Jolly-Breithaupt et al. [2] conducted a metabolism trial to determine the effects of corn oil removal from MDGS (de-oiled) on nutrient digestibility and ruminal pH, reporting that oil removal had no impact on DM, OM, or NDF digestibility. Average ruminal pH was lower for steers fed de-oiled MDGS than for steers fed normal-fat MDGS [3].

Although the practice of adding corn oil to the diet is a common feedlot practice and previous literature has evaluated the differences in cattle performance and digestibility between de-oiled versus normal-fat DGS, there is no previous literature that evaluates the removal of corn oil from DGS and the impact on cattle performance when corn oil is added back to a diet containing de-oiled distillers grains. The objectives of these experiments were to determine the effects of the removal of corn oil from MDGS and the replacement with supplemental corn oil on finishing cattle performance and total tract digestibility.

## 2. Materials and Methods

All animal care and management procedures were approved by the University of Nebraska-Lincoln Institution of Animal Care and Use Committee (IACUC #1282) approved on 12 April 2016.

### 2.1. Experiment 1

A 134 d finishing experiment conducted at the Eastern Nebraska Research, Extension, and Education Center near Mead, NE, utilized 320 crossbred yearling steers (initial BW = 413 kg ± 25 kg) in an unstructured treatment design.

Prior to experiment initiation, steers were utilized in a receiving study that evaluated different BRD vaccinations [4]. Initial processing included vaccination with a modified live viral vaccine (Titanium 5 or Titanium 5 PH-M; Elanco Animal Health, Greenfield, IN, USA), if cattle received Titanium 5, they were given a *Mannheimia haemolytica* vaccine to provide a similar immune response to Titanium 5 PH-M (Nuplura PH; Elanco Animal Health). All cattle received a *Histophilus somni* vaccine (Somnu Shield; Elanco Animal Health), and were administered an injectable dewormer (Dectomax Injectable; Zoetis Animal Health, Parsippany, NJ, USA). Cattle were revaccinated 33 d later with a modified live viral vaccine (Titanium 5; Elanco Animal Health) and a vaccine for the prevention of seven clostridial diseases (Ultrabac 7; Zoetis Animal Health). Cattle were implanted with 200 mg trenbolone acetate + 20 mg estradiol + 29 mg tylosin tartrate (Component TE-200^®^; Elanco Animal Health) 104 d prior to harvest.

Five days prior to experiment initiation, cattle were limit-fed a diet of 50% alfalfa and 50% Sweet Bran (DM-basis) offered at 2% of BW to reduce variation in gastrointestinal fill [5]. Cattle were weighed on d 0 and 1 to establish an accurate initial BW [6]. Steers were stratified into three blocks according to initial BW and assigned randomly to a pen within the block. Block one included two reps, block two included five reps, and block three included one rep. A total of 32 pens were used with 10 steers per pen. Pens were assigned randomly to one of four treatments (eight pens per treatment). All cattle were adapted to their respective finishing treatment diet over a five-step adaptation process by replacing alfalfa with dry-rolled corn (DRC) and high-moisture corn (HMC) over a 21 day period. All treatments had the same decrease in alfalfa hay with adaptation diets consisting of 42.5, 32.5, 22.5, 12.5, then 3.5% for the finishing diet. The three treatments that contained MDGS included it at respective inclusion levels throughout the step-up period and corn oil was included in the MDGS + Oil treatment throughout the step-up period as well.

The four treatments consisted of a dry-rolled corn (DRC) and high-moisture corn (HMC) blend control diet (CON), 40% de-oiled MDGS (DODGS), 40% full-fat MDGS (FFDGS), or 38% de-oiled MDGS plus 2% corn oil (DODGS + Oil) formulated to equal the fat content of FFDGS with all values reported on a DM basis (Table 1). The de-oiled MDGS (DODGS) contained 8.9% fat and the full-fat MDGS (FFDGS) contained 11.6% fat. All byproducts used in the experiment were sourced from the same ethanol plant (E Energy Adams, Adams, NE, USA). Although the DODGS + Oil and FFDGS treatments were formulated to have equal fat content; actual analysis showed the DODGS + Oil treatment contained 7.78% dietary fat and the FFDGS treatment contained 7.10% dietary fat. On a DM basis, all four treatment diets contained 3.5% alfalfa hay, 4.0% sorghum silage, 5.0% supplement, and a 50:50 blend of DRC:HMC to make up the remainder of the diet. The supplement for the CON treatment contained 2.0% Empyreal corn gluten meal (Cargill, Blair, NE, USA) for d 1–50 then 1.0% Empyreal for days 51–85 to meet metabolizable protein requirements [7]. Empyreal corn gluten meal is 75% CP with 65% of CP in the form of rumen undegradable protein (RUP), making Empyreal an excellent source of RUP for cattle. Using Empyreal corn gluten meal as an RUP source in CON treatment ensured that any performance improvement from the MDGS treatments was not due to a protein response between treatments. Empyreal corn gluten meal was removed from the CON supplement after d 85 as the need for RUP supplementation decreases as cattle grow and was based on NRC model predictions [7] to meet the metabolizable protein requirements. Urea was included in the control diet at 1.52% of DM, but is unnecessary in diets with MDGS at 30% inclusion [8]. The supplement provided tylosin phosphate (Tylan-40^®^; Elanco Animal Health) at 90 mg per steer daily and monensin (Rumensin-90^®^; Elanco Animal Health) at 33.1 g per metric ton DM.

Feed bunks were assessed at approximately 0600 h and managed to contain trace amounts (≤0.20 kg) of feed at time of feeding. Refused feed was removed, weighed, and dried in a forced air oven for 48 h at 60 °C for DM determination ([9]; Method 930.15) to calculate accurate DMI. Diets were mixed and delivered daily at approximately 0800 h using a truck-mounted feed mixer and delivery unit (Roto-Mix model 274, Roto-Mix, Dodge City, KS, USA). Individual ingredient samples were taken weekly and analyzed for DM content. Experiment 2 was conducted during part of the same time period as Experiment 1, so ingredient samples from Exp. 2 were used for Exp. 1 nutrient analysis. Ingredient samples were taken on days 9 and 12 of each period in Exp. 2 and composited by period, lyophilized, ground through a 1 mm screen of a Wiley Mill (Thomas Scientific, Swedesboro, NJ, USA), and analyzed for DM, OM, NDF, fat, and CP. Experiment 1 was longer than Exp. 2, so for the three extra months, weekly ingredient samples were composited by month, lyophilized, ground through a 1 mm screen of a Wiley Mill, and analyzed for DM, OM, NDF, fat, CP, and sulfur. Ash and OM were determined by placing samples in a muffle furnace for 6 h at 600 °C ([10]; method 4.1.10). Fat analysis was based on ether extract determined by performing a biphasic lipid extraction procedure [1]. Samples were heated in a 1:1 mixture of hexane and diethyl ether for 9 h, dilute HCl was added, and samples were centrifuged to separate the lipid layer from other liquid. The lipid layer was pipetted off, heated to separate off the remaining solvent, and weighed. The procedure for determining NDF is outlined by Van Soest et al. [11] with modifications described by Buckner [12]. Determination of CP and S were completed using a combustion type N and S analyzer (TruSpec N Determinator and TruSpec Sulfur Add-On Module, Leco Corporation, St. Joseph, MI, USA; [10]; method 990.03).

On d 134, steers were fed 50% of the previous day’s feed call and were shipped to a commercial abattoir (Greater Omaha Packing, Omaha, NE, USA) at approximately 1700 h. On the day of harvest, hot carcass weight (HCW) and liver score were collected. Following a 48 h chill, USDA marbling score, LM area, and 12th rib fat thickness were captured via plant camera grading and were recorded at time of grading. The final BW, ADG, and G:F were calculated using HCW adjusted to a common dressing percentage of 63%. Feeding values were calculated using the following equation: [((compared treatments G:F- CON G:F)/CON G:F)/compared treatments byproduct inclusion rate ×100 + 100]. Yield grade was calculated as: 2.50 + (0.98425 × fat thickness, cm) + (0.2 × 2.5 [KPH, %]) + (0.00837 × HCW, kg) − (0.0496 × LM area, cm^2^) [13]. Dietary NEm and NEg values were calculated for each treatment based on intake and performance of steers. The data were analyzed as dietary NE for each pen, similar to performance data using equations from the NRC [7] as described by Vasconcelos and Galyean [14].

Animal performance and carcass characteristics were analyzed as an unstructured treatment design using a protected F-test, with the block included as a fixed effect. Data were analyzed using the MIXED procedure of SAS (SAS Institute, Inc. Cary, NC, USA), with the pen as the experimental unit. Treatment differences were declared significant at *p* ≤ 0.05. Three steers from the FFDGS treatment died on d 52, 121, and 125 due to heat stress and bad lungs, and one steer from the DODGS + Oil treatment died on d 130 due to heat stress. One steer from the FFDGS treatment and one steer from the DODGS treatment were removed on day 95 and 101, respectively, due to injuries. These six steers were removed from the performance data.

### 2.2. Experiment 2

A 70 d metabolism experiment utilized five ruminally fistulated crossbred yearling steers (initial BW = 542 kg ± 40 kg) in a 5 × 4 unbalanced Latin rectangle design with four periods and four treatments, at the University of Nebraska Metabolism Lab (Lincoln, NE, USA). Steers were assigned randomly to sequence whereby each steer received one of four treatments, with two steers per period on the same treatment (due to 5 steeers on study).

Dietary treatments were similar to those fed in Exp. 1 (Table 1). All byproducts utilized in the trial were sourced from the same ethanol plant (E Energy Adams, Adams, NE, USA) and had the same fat concentrations as Exp. 1. Although the DODGS + Oil and FFDGS treatments were formulated to have equal fat content, dietary analysis determined that the DODGS + Oil treatment contained 7.86% dietary fat and the FFDGS treatment contained 7.09% dietary fat. The CON treatment supplement contained 1.0% Empyreal corn gluten meal (Cargill Corn Milling) to meet the metabolizable protein requirements. Empyreal was used because it is an excellent source of RUP, as described in Exp. 1. The supplement also provided tylosin phosphate (Tylan-40^®^; Elanco Animal Health) at 90 mg per steer daily and monensin (Rumensin-90^®^; Elanco Animal Health) at 30 g per ton DM. Steers were previously on a high-grain diet, so they were adapted to treatments by blending the two diets over a four-step process across 4 days (old diet, new diet; 3/4, 1/4, 1 d; 1/2, 1/2, 2 d; 1/4, 3/4, 1 d; 0, 1).

Steers were housed in individual concrete slatted pens that were 2.1 × 3.7 m with ad libitum access to feed and water. Cattle were fed once daily at 0800 h with refused feed removed prior to each daily feeding. Refused feed was collected from d 10 to 13, frozen at −20 °C, and later a subsample was dried for 48 h in a 60 °C forced air oven to determine DM and adjust intakes. Ingredient samples were taken on d 9 and 12 of each period and composited by period. Samples were lyophilized, ground through a 1 mm screen of a Wiley Mill (Thomas Scientific, Swedesboro, NJ, USA), and analyzed for DM, OM, NDF, fat, CP, and energy through bomb calorimetry to calculate nutrient composition of dietary treatments (Table 1). Nutrient analysis was completed with the same lab procedures as outlined in Exp. 1.

Each period was 14 d, which consisted of a 10-d adaptation phase and 4-d collection phase. Titanium dioxide, an indigestible marker, was dosed intraruminally twice daily at 0800 and 1600 h throughout the entire period to provide a total of 10 g/d for use as an estimate of fecal output [15]. On d 10 to 13, fecal grab samples were collected four times/d at 0800, 1200, 1600, and 2000 h, and immediately frozen at −20 °C. At the end of each period, fecal samples were composited by day (wet basis), lyophilized, and ground through a 1 mm screen of a Wiley Mill (Thomas Scientific), and composited by period. Fecal sample analysis consisted of DM, OM, NDF, fat, energy through bomb calorimetry, and titanium dioxide using the procedure described by Myers et al. [15], then plated, and analyzed using a SpectraMAX 250 (Harlow Scientific, Arlington, MA, USA).

Submersible wireless pH probes (Dascor, Inc., Escondido, CA, USA) were placed in the rumen for the entire period; however, ruminal pH was only analyzed from d nine to d 12. Ruminal pH measurements were recorded every minute (1440 measurements/d) and downloaded on d 14 of each period. Probes were attached to a weight to ensure the electrode remained submerged in the rumen contents. All probes were calibrated prior to placement in the rumen and after removal by submersing them in pH 4 and 7 standard solutions. Measurements were adjusted using the beginning and ending calibration values. Measurements for pH include average ruminal pH, minimum and maximum pH, and magnitude of pH change.

Amounts of approximately 250–300 g of whole rumen contents were collected on d 14 at 1300 h from each of the five steers on trial. Contents were placed in 1000 mL ANKOM RF Gas Production System bottles (ANKOM Technology, Macedon, NY, USA) and weights were recorded. Two gas production bottles were used for each steer. Gas production modules were placed on the bottles and the bottles were put into a 39 °C water bath. The pressure, in psi, was measured every 30 min for six hours, and measurements were automatically recorded. Pressure measurements were adjusted for pressure of an empty bottle that was used as a blank, DM of whole rumen contents, and amount of DM that was put into the bottles. Total mL gas produced per g of DM was calculated, which was then run through the NLIN procedure of SAS to determine treatment means for total gas produced and rate in %/h. Treatment means were then analyzed using the MIXED procedure of SAS to determine treatment differences.

Production of VFA was calculated over the six-hour gas production period. Two 250 mL bottles (0 h) were filled with whole rumen contents when other rumen samples were taken and frozen in liquid nitrogen. After the gas run, contents of ANKOM bottles were emptied into 250 mL bottles (6-h) and frozen in liquid nitrogen. Concentrations of VFAs were measured on the zero- and six-hour bottles. The difference in VFA production between the two time points was calculated and then divided by six hours to obtain the VFA production rate in mM/hr.

Dietary NEm and NEg values were calculated for each treatment based on DE (Mcal/d) values using equations from *Nutrient Requirements of Beef Cattle* [16]. First, metabolizable energy (ME; Mcal/d) was calculated from DE, with the following equation: ME = DE × 0.82. Concentration of ME (Mcal/kg) was calculated with the following equation: [ME] = ME/DMI (kg/d). Dietary NEm (Mcal/kg) was calculated as NEm = 1.37 × [ME] − 0.138 × [ME]^2^ + 0.0105 × [ME]^3^ − 1.12. Dietary NEg (Mcal/kg) was calculated as NEg = 1.42 × [ME] − 0.174 × [ME]^2^ + 0.0122 × [ME]^3^ − 1.65.

Digestibility, intake, and in situ data were analyzed using the MIXED procedure of SAS (SAS Institute, Inc. Cary, NC, USA). The fixed effects in the model were treatment and period, while steer was a random effect. Ruminal pH data were summarized as a repeated measure by hour and analyzed using the GLIMMIX procedure of SAS (SAS Institute, Inc.), but the day was not repeated, so hour data are for each period by steer. Slope of VFA production was analyzed using the MIXED procedure of SAS, with steer being a random effect. Treatment effects were evaluated using the F-test statistic and assessed as significant at *p* ≤ 0.05. If significant, then treatments were separated and compared using a *t*-test.

## 3. Results and Discussion

### 3.1. Experiment 1

Initial BW (*p* = 0.43; Table 2) was not influenced by treatment, based on allocation. Intakes were impacted by treatment (*p* < 0.01) with steers fed DODGS having the greatest DMI (10.8 kg) and all other treatments being similar in DMI. Dietary treatment tended to impact ADG (*p* < 0.06), with DODGS and DODGS + Oil having the greatest ADG (1.68 and 1.65 kg, respectively); FFDGS-fed cattle had intermediate ADG (1.61 kg), while CON was lowest (1.52 kg). Jolly-Breithaupt et al. [2] observed greater ADG for cattle fed de-oiled WDGS compared to full-fat WDGS (*p* < 0.01). The results for DMI and ADG from the current experiment do not agree with Bremer [17] who observed no difference in DMI or ADG between de-oiled and full-fat MDGS. Vander Pol et al. [3] observed a decrease in DMI and ADG as supplemental corn oil was added to a corn-based diet. As a result of increased ADG, G:F was impacted by treatment (*p* < 0.01), with DODGS + Oil steers having the greatest G:F, although not differing from the FFDGS treatment, and CON steers having the lowest G:F. Feed efficiency was improved by 1.2% for steers fed the FFDGS treatment compared to the DODGS treatment. Bremer [17] reported that cattle fed full-fat MDGS at 30% of diet DM were 3.4% more efficient than steers fed 30% de-oiled MDGS. In the current experiment, when 2% corn oil was added back to de-oiled MDGS (DODGS + Oil), there was a 4.9% improvement in G:F compared to DODGS. The feeding value of DODGS was calculated to be 115% of corn and FFDGS was 119% the feeding value of corn. These results differ from Jolly-Breithaupt et al. [2] who reported a feeding value of 130% of corn for both de-oiled and normal-fat MDGS at 40% diet DM inclusion. Bremer [17] observed a feeding value of 117% of corn for de-oiled MDGS at 30% diet DM inclusion, which is similar to the current experiment; however, they observed 129% as the feeding value of corn for full-fat MDGS, which is greater than the current experiment. Dietary treatment impacted NEm and NEg (*p* < 0.01), with DODGS + Oil having the greatest NE values and CON having the lowest (Table 2). Bremer [17] also reported tendencies for NEm and NEg to be greater for full-fat MDGS compared to de-oiled MDGS at 30% diet DM inclusion level. However, the values in the current experiment were higher than what Bremer [17] observed. There was no difference between the NEm value for DODGS + Oil when compared to FFDGS, but DODGS + Oil had greater NEg than all other treatments.

Hot carcass weight (HCW) differed between treatments (*p* = 0.05) with steers on DODGS treatment having the heaviest HCW (406 kg) though not much different from DODGS + Oil (404 kg) or FFDGS (401 kg), and steers in the CON treatment having the lightest HCW (Table 2). Dietary treatment did not impact marbling score (*p* = 0.64) or LM area (*p* = 0.52), with steers averaging a marbling score of 459, qualifying for USDA choice quality grade, and LM area of 87.8 cm^2^. Dietary treatment impacted 12th rib fat thickness (*p* = 0.01) and calculated YG (*p* = 0.02), where the three treatments that included MDGS (DODGS; DODGS + Oil; FFDGS) had similar fat thickness and calculated YG and were greater than that of the corn CON. The increase in calculated YG for all three treatments that included MDGS in the diet was observed due to the increased 12th rib fat thickness and heavier HCW while no difference was observed in LM area compared to CON treatment. Jolly-Breithaupt et al. [2] and Bremer [17] reported that carcass characteristics were not impacted by the removal of oil from distillers grains compared to full-fat distillers grains plus solubles.

### 3.2. Experiment 2

Dry matter intake was not impacted by dietary treatment (*p* = 0.94; Table 3) with intakes ranging from 8.9 kg/d for CON to 9.4 kg/d for FFDGS. These results differed from Vander Pol et al. [3] and Bremer et al. [18], who observed a reduction in DMI when corn oil was added to feedlot diets. Intakes observed in Exp. 2 were numerically lower than what was observed in Exp. 1 (8.9 vs. 10.3 kg for CON, 9.3 vs. 10.8 kg for DODGS, 9.0 vs. 10.0 kg for DODGS + Oil, 9.4 vs. 10.2 kg for FFDGS). Dietary treatment had an impact on total tract DM digestibility (*p* < 0.01), where the greatest DM digestibility was observed for CON treatment and differed from all other dietary treatments. Total tract DM digestibility results agree with Corrigan et al. [19], Vander Pol et al. [3], and Bremer et al. [18], who reported that including DGS in the diet reduced DM digestibility when compared to feeding corn control diets with no DGS. They also noted that DM digestibility is reduced when corn oil is added to the diet. Results of OM intake (kg/d) and total tract digestibility followed the same trend as DM, where OM intake did not differ between dietary treatments (*p* = 0.96) and total tract OM digestibility differed between treatments (*p* < 0.01) with the corn CON treatment having the greatest OM digestibility. Jolly-Breithaupt et al. [2] compared de-oiled MDGS and normal-fat MDGS and reported no differences in DM or OM intake and total tract digestibility.

A treatment effect was observed for NDF intake (*p* < 0.01), with treatments containing MDGS (FFDGS; DODGS; DODGS + Oil) having greater NDF intake than CON (Table 3). Greater NDF intake is due to greater NDF concentration in diets that contain DGS as DGS are approximately 33.7% NDF on a DM basis while dry-rolled corn is approximately 9.7% on a DM basis [16]. There was a tendency (*p* = 0.07) for total tract NDF digestibility to differ among treatments, with the greatest NDF digestibility observed for FFDGS and the lowest for CON and DODGS + Oil, with DODGS being intermediate and similar to all other treatments. Corrigan et al. [19] and Bremer et al. [18] also reported lower NDF digestibility for corn control treatments when compared to dietary treatments that included MDGS. Vander Pol et al. [3] observed that total tract DM, OM, and NDF digestibility were less (*p* < 0.10) for cattle fed a composite diet consisting of corn bran and corn gluten meal and a composite diet plus oil when compared to WDGS, a corn control diet, and a corn control diet plus oil. Vander Pol et al. [3] concluded that equal amounts of fat provided from WDGS or corn oil do not result in similar impacts on NDF digestion, with added oil having a negative impact on NDF digestion compared to oil that is naturally present in distillers grains. Bremer et al. [18] fed diets containing 8.5% lipid inclusion from varying fat sources and concluded that diets containing distillers grains that supplied lipid up to 8% of diet DM can be fed to cattle without having a negative impact on performance; however, 8% dietary lipid in the form of corn oil will have a negative impact on cattle performance. Digestion data from OM and DE are not consistent with the observed performance in Exp. 1 between full-fat MDGS (FFDGS) and adding corn oil back to de-oiled MDGS (DODGS + Oil). Results from the current experiment suggest that corn oil has a negative impact on NDF digestibility, which could be because corn oil is considered a free oil, which is available for biohydrogenation, or the feed particles could be coated with oil [20]. Free oil can therefore impact fiber digestion in the rumen, while the fat in DGS is bound in the germ, so DGS feedstuffs will pass through the rumen without negatively impacting rumen fiber digestion.

Dietary fat intake (Mcal/d) was different among dietary treatments (*p* < 0.01), with DODGS + Oil being greatest, FFDGS being an intermediate and similar to both DODGS + Oil and DODGS, and CON being the least (Table 3). There was no treatment effect observed for total tract fat digestibility (*p* = 0.83), with an observed range of 81.1 to 83.3%. Bremer et al. [18] observed values greater than 90% for total tract fat digestibility for treatments that contained varying fat sources, including corn oil, beef tallow, condensed corn distillers solubles, and WDGS. Jolly-Breithaupt et al. [2] also reported values around 90% for total tract fat digestibility, observing no difference between de-oiled and normal-fat MDGS which agrees with the results from the current study.

Energy intake (Mcal/d) and digestible energy (DE; Mcal/d) were not impacted by treatment (*p* ≥ 0.46; Table 3). Energy intake ranged from 38.6 to 45.0 Mcal/d, while DE ranged from 30.97 to 34.31 Mcal/d. Digestible energy measured as Mcal/kg did not differ between treatments (*p* = 0.13) and averaged 3.57 Mcal/kg. Wilson et al. [21] reported a DE (Mcal/kg) value of 3.68 for a 40% WDGS diet, which was similar to the current experiment. Wilson et al. [21] concluded that there was an additional supply of DE when diets contain DGS compared to a corn-based diet, which is likely due to the higher protein and fat content of DGS. The increased supply of DE in diets containing DGS is a new concept and has not been researched in detail. This concept could help explain the improvement in cattle performance from including DGS in the diet. The DE results from Exp. 2 do not match the performance results from Exp. 1, where cattle fed DODGS + Oil were numerically the most efficient and had the greatest ADG.

Dietary NE values that were calculated from NASEM [16] equations using DE from Exp. 2 were similar to values calculated from performance characteristics in Exp. 1 which used dietary NE equations from the NRC [7], except for net energy values for the DODGS + Oil treatment (Table 4). The difference observed between the NE values calculated from Exp. 1 and Exp. 2 for the DODGS + Oil treatment are proposed to be due to a difference in the previously assumed 82% conversion of DE to ME. Through back calculations, a DE to ME conversion of 87% was calculated for the DODGS + Oil treatment. Although NE values from Exp. 1 are assumed to be more correct than values from Exp. 2, there is confidence that DE values from Exp. 2 are correct due to the similarity between NE values for CON, DODGS, and FFDGS dietary treatments.

Dietary treatment did not impact ruminal pH average, maximum, minimum, or magnitude of change (*p* ≥ 0.14; Table 5). Jolly-Breithaupt et al. [2] observed an increase in average ruminal pH for cattle fed normal-fat MDGS compared to de-oiled MDGS. Bremer et al. [18] reported that ruminal pH was greatest for cattle fed a diet containing corn oil, which was similar to the numerical results observed in the current study.

The total VFA production rate (mM/hr) was impacted by dietary treatment (*p* < 0.01), with DODGS having the greatest total VFA production differing from the other three treatments (Table 6). The production rate of acetate and butyrate did not differ among treatments (*p* ≥ 0.40). Propionate production was greatest for DODGS (*p* < 0.01), intermediate for CON and DODGS + Oil, and lowest for FFDGS. The total VFA production agrees with observed pH data, where DODGS + Oil and FFDGS had numerically greater pH and a lower rate of production, while CON and DODGS had numerically lower pH with a greater rate of VFA production. There were no hour × treatment interactions for molar proportion of VFA (*p* ≥ 0.96). The molar proportion of acetate was impacted by dietary treatment (*p* < 0.01) with FFDGS having the greatest acetate molar production, DODGS and DODGS + Oil having the least, with CON as an intermediate and similar to all other treatments. There was a tendency for the molar proportion of propionate (*p* = 0.06) to differ between dietary treatments, with DODGS and DODGS + Oil having the greatest molar propionate production, and FFDGS having the least. There was no dietary treatment effect observed for molar proportion of butyrate (*p* = 0.57). The acetate/propionate (A:P) molar proportion tended to differ between dietary treatments (*p* = 0.07) and was greatest for the cattle fed FFDGS and least for the cattle fed DODGS and DODGS + Oil. Similar to the results of the current experiment, Carlson [22] tested a corn-based control compared to a diet with 40% inclusion of MDGS on a DM basis and observed no differences in VFA molar proportion. Ham et al. [23] tested a DRC-based control diet compared to a diet with 40% WDGS on a DM basis and observed no difference in the amounts (mM) of acetate, propionate, butyrate, or A:P.

Total gas production was impacted by dietary treatment (*p* = 0.02), with FFDGS and DODGS + Oil having the greatest total gas production, DODGS having the least total gas production, and CON as an intermediate and similar to all treatments. The rate of gas production (%/hr) was impacted by dietary treatment (*p* = 0.03), with DODGS + Oil having the greatest rate of gas production, DODGS and FFDGS having the least total gas production, and CON as an intermediate and similar to all other treatments.

## 4. Conclusions

Partially removing oil from modified distillers grains plus solubles did not significantly impact cattle performance, carcass traits, energy content, or digestibility when MDGS was included at approximately 40% of diet DM. Including MDGS in the feedlot diet improved G:F up to 11%, impacted ADG, and increased HCW up to 3.3% when compared to a DRC:HMC blend control diet. The greatest feed efficiency was observed for steers fed DODGS + Oil and FFDGS, which supports the understanding that fat in a feedlot diet increases dietary energy intake, and, therefore, improves feed efficiency. The addition of corn oil to de-oiled MDGS (DODGS + Oil) negatively impacted OM and fiber digestion. In conclusion, the impact of de-oiling DGS during ethanol production, therefore reducing the fat content of distillers grains down to 8.9% fat (DM-basis) compared to full-fat distillers grains with 11.6% fat (DM-basis), had a negligible impact on cattle performance and carcass characteristics.

## Figures and Tables

**Table 1:** Composition (% of diet DM) of dietary treatments fed to yearling steers (Exp. 1) and steers used in the digestion experiment (Exp. 2).

Ingredient	Treatment ^1^			
	CON	DODGS	DODGS + Oil	FFDGS
Dry-rolled corn	43.75	23.75	23.75	23.75
High-moisture corn	43.75	23.75	23.75	23.75
MDGS De-oiled ^2^	-	40	38	-
MDGS Full Fat ^3^	-	-	-	40
Corn Oil	-	-	2	-
Alfalfa hay	3.5	3.5	3.5	3.5
Sorghum Silage	4	4	4	4
Supplement ^4^	-	-	-	-
Fine-Ground Corn	0.773	2.787	2.787	2.787
Limestone	1.729	1.697	1.697	1.697
Tallow	0.125	0.125	0.125	0.125
Urea	1.517	-	-	-
Potassium Chloride	0.465	-	-	-
Salt	0.3	0.3	0.3	0.3
Beef Trace Minerals	0.05	0.05	0.05	0.05
Vitamin A-D-E	0.015	0.015	0.015	0.015
Monensin premix ^5^	0.017	0.017	0.017	0.017
Tylosin premix ^6^	0.009	0.009	0.009	0.009
Analyzed Nutrient Composition, % of DM				
DM	78.5	67.0	68.0	67.3
OM	96.0	95.2	93.3	95.3
CP	12.1	17.0	16.4	16.7
NDF	11.1	22.7	22.0	22.8
Fat (Exp. 1/Exp. 2)	3.89/4.16	5.96/6.04	7.78/7.86	7.10/7.09
Sulfur	0.15	0.45	0.43	0.48

^1^ Treatments included CON = corn control (high-moisture corn: dry-rolled corn blend); DODGS = de-oiled modified distillers grains plus solubles at 40% diet DM; DODGS + Oil = de-oiled modified distillers grains plus solubles at 38% diet DM plus 2% corn oil DM-basis; FFDGS = full-fat modified distillers grains plus solubles at 40% diet DM. ^2^ DODGS: de-oiled modified distillers grains plus solubles containing 8.9% fat DM-basis. ^3^ FFDGS: full-fat modified distillers grains plus solubles containing 11.6% fat DM-basis. ^4^ Supplement fed at 5% of dietary DM. ^5^ Premix contained 198 g of monensin/kg (Rumensin-90^®^; Elanco Animal Health). ^6^ Premix contained 88 g of tylosin phosphate/kg (Tylan-40^®^; Elanco Animal Health).

**Table 2:** The effect of feeding de-oiled MDGS at 40% diet DM, full-fat MDGS at 40% diet DM, de-oiled MDGS at 38% diet DM plus 2% corn oil added back to the diet, or a corn control diet on feedlot performance and carcass characteristics (Exp. 1).

	Treatment ^1^						
	CON	DODGS	DODGS + Oil	FFDGS	SEM	F-TEST	
Feedlot Performance							
Initial BW, kg	419	420	420	420	0.5	0.43	
Final BW ^2^, kg	624 ^b^	645 ^a^	640 ^a^	636 ^ab^	5.6	0.04	
DMI, kg/d	10.3 ^b^	10.8 ^a^	10.0 ^b^	10.2 ^b^	0.15	0.01	
ADG, kg	1.52 ^b^	1.68 ^a^	1.65 ^a^	1.61 ^ab^	0.041	0.06	
G:F	0.148 ^c^	0.157 ^b^	0.165 ^a^	0.159 ^ab^	0.0028	<0.01	
Feeding value ^3^	-	115	-	119	-	-	
NEm Mcal/kg ^4^	1.96 ^c^	2.00 ^bc^	2.09 ^a^	2.05 ^ab^	0.019	<0.01	
NEg Mcal/kg ^4^	1.31 ^c^	1.34 ^bc^	1.43 ^a^	1.38 ^b^	0.017	<0.01	
Carcass Characteristics							
HCW, kg	393 ^b^	406 ^a^	404 ^a^	401 ^ab^	3.5	0.05	
Marbling ^5^	463	458	446	467	12.9	0.64	
LM area, cm^2^	87.7	88.4	88.4	86.5	1.29	0.52	
12th rib fat, cm	1.19 ^b^	1.42 ^a^	1.37 ^a^	1.40 ^a^	0.051	0.01	
Calculated YG ^6^	3.10 ^b^	3.43 ^a^	3.34 ^a^	3.44 ^a^	0.086	0.02	

^a–c^ Means with different subscripts differ (*p* < 0.05) ^1^ Treatments included CON = corn control (high-moisture corn: dry-rolled corn blend); DODGS = de-oiled modified distillers grains plus solubles at 40% diet DM; DODGS + Oil = de-oiled modified distillers grains plus solubles at 38% diet DM plus 2% corn oil DM-basis; FFDGS = full-fat modified distillers grains plus solubles at 40% diet DM. ^2^ Calculated from HCW divided by a common dressing percentage of 63.0% ^3^ Feeding value calculation: [((compared treatments G:F − CON G:F)/CON G:F)/compared treatments’ byproduct inclusion rate × 100 + 100]. ^4^ Dietary NE equations from the NRC [7] as described by Vasconcelos and Galyean [14]. ^5^ Marbling score: 400 = Slight^00^, 450 = Slight^50^, 500 = Small^00^, 550 = Small^50^; ^6^ USDA Calculated Yield Grade = 2.50 + (0.9843 × fat thickness, cm) + (0.2 × KPH, %) + (0.0084 × HCW, kg) − (0.0496 × LM area, cm^2^) [13].

**Table 3:** The effect of feeding de-oiled MDGS at 40% diet DM, full-fat MDGS at 40% diet DM, de-oiled MDGS at 38% diet DM plus 2% corn oil added back to the diet, or a corn control diet on digestible energy and intake and total tract digestibility of DM, OM, NDF, and fat (Exp. 2).

	Treatment ^1^					
	CON	DODGS	DODGS + Oil	FFDGS	SEM	F-TEST
DM						
Intake, kg/d	8.9	9.3	9.0	9.4	0.88	0.94
Digestibility, %	81.7 ^a^	77.2 ^b^	73.8 ^c^	75.9 ^bc^	1.28	<0.01
OM						
Intake, kg/d	8.6	8.8	8.6	9.0	0.84	0.96
Digestibility, %	83.6 ^a^	79.1 ^b^	76.1 ^c^	78.1 ^bc^	1.43	<0.01
NDF						
Intake, kg/d	0.98 ^b^	2.14 ^a^	1.99 ^a^	2.17 ^a^	0.174	<0.01
Digestibility, %	50.5 ^b^	55.3 ^ab^	51.3 ^b^	57.7 ^a^	2.19	0.07
Fat						
Intake, kg/d	0.37 ^c^	0.56 ^b^	0.71 ^a^	0.67 ^ab^	0.056	<0.01
Digestibility, %	82.9	81.1	81.8	83.3	1.91	0.83
Energy						
Intake, Mcal	38.6	43.3	43.0	45.0	4.08	0.46
DE, Mcal/d	30.97	33.27	31.70	34.31	2.920	0.76
DE, Mcal/kg	3.50	3.60	3.52	3.66	0.067	0.13

^a–c^ Means with different subscripts differ (*p* < 0.05) ^1^ Treatments included CON = corn control (high-moisture corn: dry-rolled corn blend); DODGS = de-oiled modified distillers grains plus solubles at 40% diet DM; DODGS + Oil = de-oiled modified distillers grains plus solubles at 38% diet DM plus 2% corn oil DM-basis; FFDGS = full-fat modified distillers grains plus solubles at 40% diet DM.

**Table 4:** The effect of feeding de-oiled MDGS at 40% diet DM, full-fat MDGS at 40% diet DM, de-oiled MDGS at 38% diet DM plus 2% corn oil added back to the diet, or a corn control diet on dietary net energy values.

	Treatment ^1^					
	CON	DODGS	DODGS + Oil	FFDGS	SEM	F-TEST
NEm Mcal/kg^2^	1.96 ^c^	2.00 ^bc^	2.09 ^a^	2.05 ^ab^	0.019	<0.01
NEm Mcal/kg^3^	1.92	1.99	1.94	2.03	-	-
NEg Mcal/kg ^2^	1.31 ^c^	1.34 ^bc^	1.43 ^a^	1.38 ^b^	0.017	<0.01
NEg Mcal/kg ^3^	1.28	1.34	1.29	1.37	-	-

^a–c^ Means with different subscripts differ (*p* < 0.05) ^1^ Treatments included CON = corn control (high-moisture corn: dry-rolled corn blend); DODGS = de-oiled modified distillers grains plus solubles at 40% diet DM; DODGS + Oil = de-oiled modified distillers grains plus solubles at 38% diet DM plus 2% corn oil DM-basis; FFDGS = full-fat modified distillers grains plus solubles at 40% diet DM. ^2^ Dietary NE equations from NRC [7] as described by Vasconcelos and Galyean [14]. ^3^ Dietary NE calculated from DE using equations from NASEM [16].

**Table 5:** The effect of feeding de-oiled MDGS at 40% diet DM, full-fat MDGS at 40% diet DM, de-oiled MDGS at 38% diet DM plus 2% corn oil added back to the diet, or a corn control diet on ruminal pH metrics (Exp. 2).

	Treatment ^1^					
	CON	DODGS	DODGS + Oil	FFDGS	SEM	Trt
Average pH	5.64	5.70	5.88	5.83	0.138	0.14
Maximum pH	6.46	6.53	6.66	6.66	0.150	0.38
Minimum pH	5.03	5.06	5.22	5.18	0.120	0.36
pH magnitude	1.43	1.47	1.45	1.49	0.112	0.97

^1^ Treatments included CON = corn control (high-moisture corn: dry-rolled corn blend); DODGS = de-oiled modified distillers grains plus solubles at 40% diet DM; DODGS + Oil = de-oiled modified distillers grains plus solubles at 38% diet DM plus 2% corn oil DM-basis; FFDGS = full-fat modified distillers grains plus solubles at 40% diet DM.

**Table 6:** The effect of feeding de-oiled MDGS at 40% diet DM, full-fat MDGS at 40% diet DM, de-oiled MDGS at 38% diet DM plus 2% corn oil added back to the diet, or a corn control diet on VFA production (mM/hr), VFA molar proportion, amount of ruminal gas produced, and rate of production (%/hr) (Exp. 2).

	Treatment ^1^					
	CON	DODGS	DODGS + Oil	FFDGS	SEM	*p*-Value
VFA production						
Total, mM	13.6 ^b^	17.2 ^a^	12.5 ^b^	11.2 ^b^	1.74	<0.01
Acetate	5.4	6.8	5.4	5.1	0.88	0.40
Propionate	5.7 ^b^	7.7 ^a^	4.8 ^bc^	3.8 ^c^	0.87	<0.01
Butyrate	1.7	1.9	1.8	1.8	0.35	0.99
VFA molar %						
Acetate	48.1 ^ab^	45.4 ^b^	45.1 ^b^	51.6 ^a^	1.49	0.01
Propionate	35.1	38.3	37.0	29.7	2.30	0.06
Butyrate	12.1	12.1	13.9	13.7	1.21	0.57
A:P ^2^	1.6 ^ab^	1.3 ^b^	1.3 ^b^	1.9 ^a^	0.23	0.07
Gas production						
Total	8.52 ^ab^	7.31 ^b^	9.19 ^a^	9.61 ^a^	0.813	0.02
Rate (%/hr)	54.9 ^ab^	51.5 ^b^	59.1 ^a^	51.0 ^b^	2.88	0.03

^a–c^ Means with different subscripts differ (*p* < 0.05) ^1^ Treatments included CON = corn control; DODGS = 40% de-oiled modified distillers grains plus solubles; DODGS + Oil = 38% de-oiled modified distillers grains plus solubles plus 2% corn oil; FFDGS = 40% full-fat modified distillers grains plus solubles. ^2^ A:P = Acetate/propionate ratio.

## Data Availability

The raw data supporting the conclusions of this article will be made available by the authors on request.
